# Forgiver Triumphs in Alternating Prisoner's Dilemma

**DOI:** 10.1371/journal.pone.0080814

**Published:** 2013-12-12

**Authors:** Benjamin M. Zagorsky, Johannes G. Reiter, Krishnendu Chatterjee, Martin A. Nowak

**Affiliations:** 1 Program for Evolutionary Dynamics, Harvard University, Cambridge, Massachusetts, United States of America; 2 IST Austria (Institute of Science and Technology Austria), Klosterneuburg, Lower Austria, Austria; 3 Department of Mathematics, Department of Organismic and Evolutionary Biology, Harvard University, Cambridge, Massachusetts, United States of America; Fondazione Edmund Mach, Research and Innovation Centre, Italy

## Abstract

Cooperative behavior, where one individual incurs a cost to help another, is a wide spread phenomenon. Here we study direct reciprocity in the context of the alternating Prisoner's Dilemma. We consider all strategies that can be implemented by one and two-state automata. We calculate the payoff matrix of all pairwise encounters in the presence of noise. We explore deterministic selection dynamics with and without mutation. Using different error rates and payoff values, we observe convergence to a small number of distinct equilibria. Two of them are uncooperative strict Nash equilibria representing always-defect (ALLD) and Grim. The third equilibrium is mixed and represents a cooperative alliance of several strategies, dominated by a strategy which we call Forgiver. Forgiver cooperates whenever the opponent has cooperated; it defects once when the opponent has defected, but subsequently Forgiver attempts to re-establish cooperation even if the opponent has defected again. Forgiver is not an evolutionarily stable strategy, but the alliance, which it rules, is asymptotically stable. For a wide range of parameter values the most commonly observed outcome is convergence to the mixed equilibrium, dominated by Forgiver. Our results show that although forgiving might incur a short-term loss it can lead to a long-term gain. Forgiveness facilitates stable cooperation in the presence of exploitation and noise.

## Introduction

A cooperative dilemma arises when two cooperators receive a higher payoff than two defectors and yet there is an incentive to defect [1], [2]. The Prisoner's Dilemma [3]–[9] is the strongest form of a cooperative dilemma, where cooperation requires a mechanism for its evolution [10]. A mechanism is an interaction structure that specifies how individuals interact to receive payoff and how they compete for reproduction. Direct reciprocity is a mechanism for the evolution of cooperation. Direct reciprocity means there are repeated encounters between the same two individuals [11]–[37]. The decision whether or not to cooperate depends on previous interactions between the two individuals. Thus, a strategy for the repeated Prisoner's Dilemma (or other repeated games) is a mapping from any history of the game into what to do next. The standard theory assumes that both players decide simultaneously what do for the next round. But another possibility is that the players take turns when making their moves [38]–[40]. This implementation can lead to a strictly alternating game, where the players always choose their moves in turns, or to a stochastically alternating game, where in each round the player to move is chosen at randomnext is selected probabilistically. Here we investigate the strictly alternating game.

We consider the following scenario. In each round a player can pay a cost, 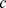, for the other player to receive a benefit, 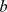, where 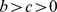. If both players cooperate in two consecutive moves, each one gets a payoff, 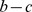, which is greater than the zero payoff they would receive for mutual defection. But if one player defects, while the other cooperates, then the defector gets payoff, 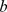, while the cooperator gets the lowest payoff, 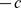. Therefore, over two consecutive moves the payoff structure is the same as in a Prisoner's Dilemma: 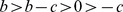. Thus, this game is called “alternating Prisoner's Dilemma” [29], [39].

**Figure 1:**
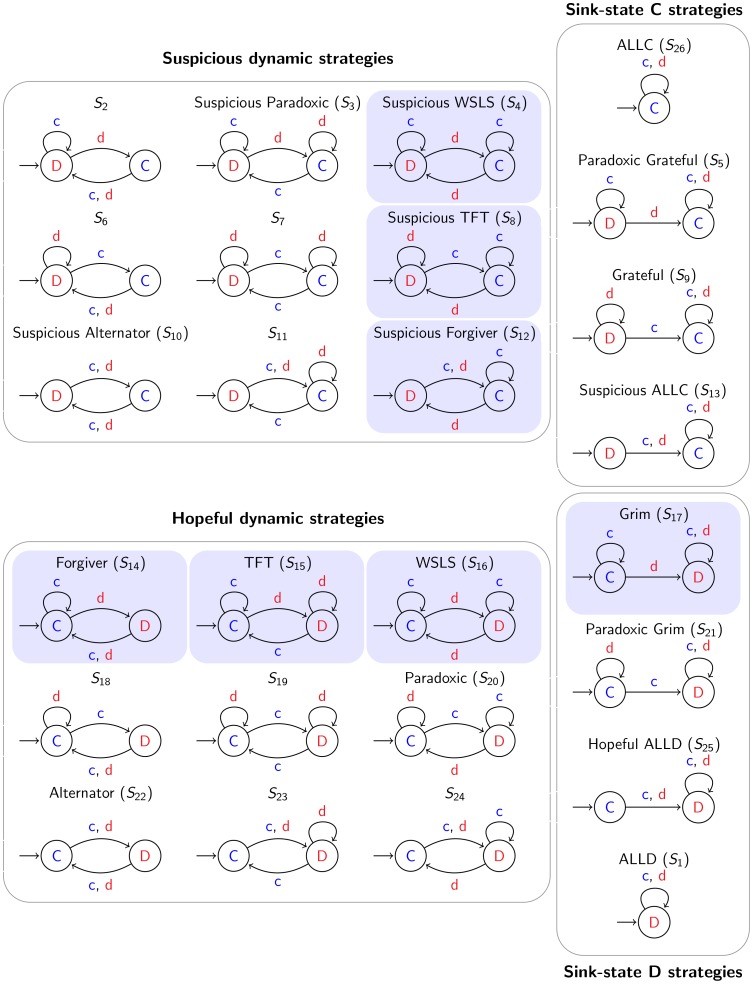
Deterministic strategies in the Prisoner's Dilemma. Each automaton defines a different strategy for how a player behaves during the game. If a player is in state 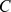, she will cooperate in the next move; if she is in state 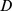, then she will defect. The outgoing transitions of a state define how the state of an automaton will change in response to cooperating (label c) or defecting (label d) of the opponent. The left state with the small incoming arrow corresponds to the initial state of a strategy. The 26 distinct strategies (automata) are classified into four categories: (i) sink-state C strategies, (ii) sink-state D strategies, (iii) suspicious dynamic strategies, and (iv) hopeful dynamic strategies. The automata with the blue background shading contain a conditional cooperation element (Fig. S1 in File S1) which ensures the benefit of mutual cooperation but also avoids being exploited by defection-heavy strategies.

We study the strictly alternating Prisoner's Dilemma in the presence of noise. In each round, a player makes a mistake with probability ε leading to the opposite move. We consider all strategies that can be implemented by deterministic finite state automata [41] with one or two states. These automata define how a player behaves in response to the last move of the other player. Thus we consider a limited strategy set with short-term memory. Finite-state automata have been used extensively to study repeated games [42]–[45] including the simultaneous Prisoner's Dilemma. In our case, each state of the automaton is labeled by 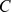 or 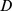. In state 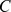 the player will cooperate in the next move; in state 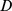 the player will defect. Each strategy starts in one of those two states. Each state has two outgoing transitions (either to the same or to the other state): one transition specifies what happens if the opponent has cooperated (labeled with 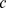) and one if the opponent has defected (labeled with 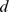). There are 26 automata encoding unique strategies (Fig. 1). These strategies include ALLC, ALLD, Grim, tit-for-tat (TFT), and win-stay lose-shift (WSLS).

ALLC (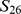) and ALLD (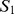) are unconditional strategies (see Fig. 1 and Supporting File S1 for strategy names and their indexing). ALLC always cooperates while ALLD always defects. Both strategies are implemented by a one-state automaton (Fig. 1). The strategy Grim starts and stays in state 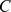 as long as the opponent cooperates. If the opponent defects, Grim permanently moves to state 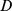 with no possibility to return. TFT (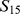) starts in state 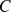 and subsequently does whatever the opponent did in the last round [5]. This simple strategy is very successful in an error-free environment as it promotes cooperative behavior but also avoids exploitation by defectors. However, in a noisy environment TFT achieves a very low payoff against itself since it can only recover from a single error by another error [46]. WSLS (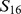) has the ability to correct errors in the simultaneous Prisoner's Dilemma [47]. This strategy also starts in state 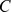 and moves to state 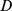 whenever the opponent defects. From state 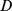 strategy WSLS switches back to cooperation only if another defection occurs. In other words, WSLS stays in the current state whenever it has received a high payoff, but moves to the other state, if it has received a low payoff.

We can divide these 26 strategies into four categories: (i) sink-state C (ssC) strategies, (ii) sink-state D (ssD) strategies, (iii) suspicious dynamic strategies, and (iv) hopeful dynamic strategies. Sink-state strategies always-cooperate or always-defect either from the beginning or after some condition is met. They include ALLC, ALLD, Grim and variations of them. There are eight sink-state strategies in total. Suspicious dynamic strategies start with defection and then move between their defective and cooperative state depending on the other player's decision. Hopeful dynamic strategies do the same, but start with cooperation. There are nine strategies in each of these two categories. For each suspicious dynamic strategy there is a hopeful counterpart.

Some of the dynamic strategies do little to optimize their score. For example, Alternator (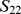) switches between cooperation and defection on each move. But a subset of dynamic strategies are of particular interest: Forgiver (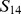), TFT, WSLS, and their suspicious counterparts (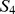, 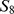, and 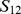). These strategies have the design element to stay in state 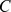 if the opponent has cooperated in the last round but move to state 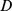 if the opponent has defected; we call this element the conditional cooperation element (see Fig. S1 in File S1). In state 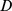, TFT then requires the opponent to cooperate again in order to move back to the cooperative state. WSLS in contrast requires the opponent to defect in order to move back to the cooperative state. But Forgiver moves back to the cooperative state irrespective of the opponents move (Fig. 1: hopeful dynamic strategies).

**Figure 2:**
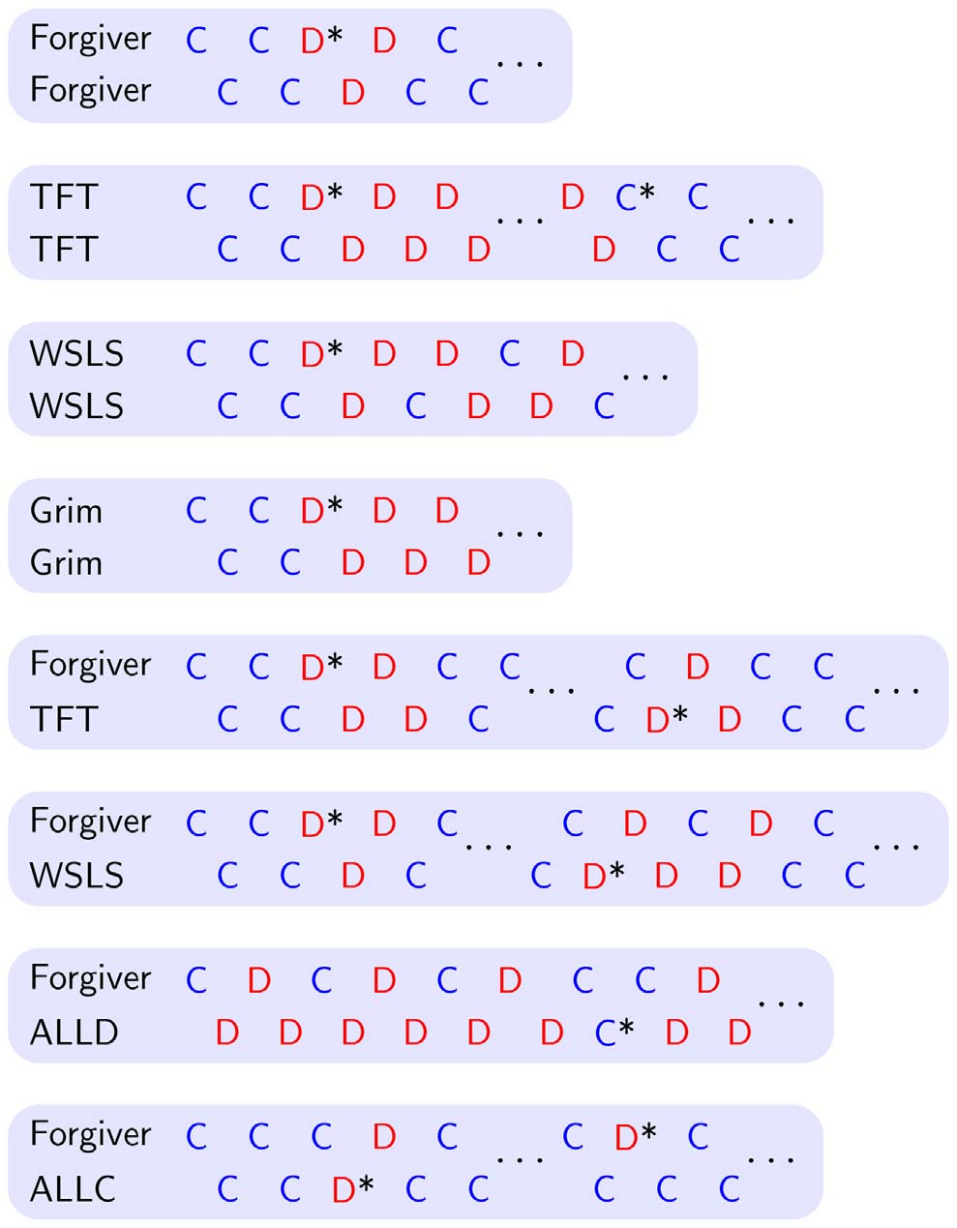
Performance of the conditional cooperators in the presence of noise. An asterisk after a move indicates that this move was caused by an error. When the conditional cooperators are playing against a copy of themselves, Forgiver performs very well as it can recover from an accidental defection within three rounds. Against defection-heavy strategies like Grim and ALLD, Forgiver gets exploited in each second round. Both TFT and WSLS are not error correcting as they are unable to recover back to cooperation after an unintentional mistake. Only another mistake can enable them to return to cooperative behavior. When Grim plays against itself and a single defection occurs, it moves to the defection state with no possibility of returning to cooperation.

Neither TFT nor WSLS are error correcting in the alternating game [29], [39]. In a game between two TFT players, if by mistake one of them starts to defect, they will continue to defect until another mistake happens. The same is true for WSLS in the alternating game. Thus WSLS, which is known to be a strong strategy in the simultaneous game, is not expected to do well in the alternating game. Forgiver, on the other hand, is error correcting in the alternating game. It recovers from an accidental defection in three rounds (Fig. 2).

A stochastic variant of Forgiver is already described in [39]. In this study, strategies are defined by a quadruple 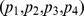 where 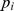 denotes the probability to cooperate after each of the four outcomes 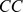, 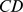, 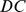, and 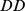. This stochastic strategy set is studied in the setting of the infinitely-repeated alternating game. The initial move is irrelevant. In [39] a strategy close to 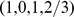 is victorious in computer simulations of the strictly alternating Prisoner's Dilemma. For further discussions see also pp. 78–80 in [29]; there the stochastic variant of Forgiver is called ‘Firm but Fair’.

## Results

We calculate the payoff for all pairwise encounters in games of 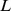 moves of both strategies, thereby obtaining a 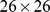 payoff matrix. We average over which strategy goes first. Without loss of generality we set 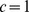. At first we study the case 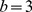 with error rate 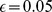 and an average game length of 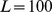. Table 1 shows a part of the calculated payoff matrix for six relevant strategies. We find that ALLD (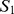) and Grim (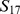) are the only strict Nash equilibria among the 26 pure strategies. ALLC (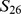) vs ALLC receives a high payoff, but so does Forgiver vs Forgiver. The payoffs of WSLS vs WSLS and TFT vs TFT are low, because neither strategy is error correcting (Fig. 2). Interestingly TFT vs WSLS yields good payoff for both strategies, because their interaction is error correcting.

**Table 1:** Payoff matrix for the most relevant strategies.

	ALLD	Forgiver	TFT	WSLS	Grim	ALLC
**ALLD**	**10.0**	148.4	24.8	144.9	11.5	280.0
**Forgiver**	−36.1	174.8	163.5	166.9	−12.7	194.3
**TFT**	5.1	178.1	104.5	176.7	24.5	194.5
**WSLS**	−35.0	169.1	162.3	106.5	−12.0	230.9
**Grim**	9.5	152.0	40.5	148.8	**28.1**	262.9
**ALLC**	−80.0	177.2	176.6	67.3	−28.8	190.0

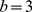
 (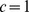), the error rate 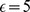
%, and the number of rounds in each game 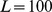. There are two pure Nash equilibria in the full payoff matrix: ALLD (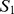) and Grim (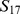), both denoted in bold. Excerpt of the payoff matrix with the most relevant strategies when the benefit value

In the following, we study evolutionary game dynamics [48]–[50] with the replicator equation. The frequency of strategy 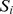 is denoted by 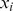. At any one time we have 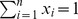, where 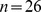 is the number of strategies. The frequency 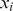 changes according to the relative payoff of strategy 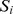. We evaluate evolutionary trajectories for many different initial frequencies. The trajectories start from 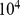 uniformly distributed random points in the 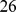-simplex.

**Figure 3:**
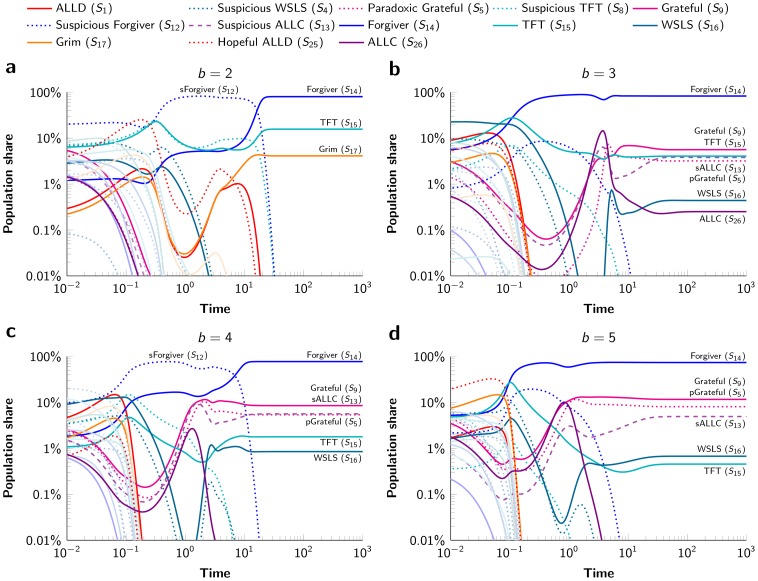
The evolution of strategies in the alternating Prisoner's Dilemma. In all panels, the simulations start from a randomly chosensome random point in the 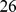-simplex. In the cases of 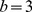, 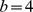, and 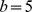, the evolutionary trajectories converge to a cooperative alliance of many strategies dominated by the strategy Forgiver. In the case of 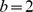, the evolutionary trajectories converge to a mixed equilibrium of Forgiver, TFT, and Grim. The error rate 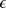 is set to 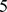
%, the number of rounds per game is 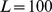, and the mutation rate is 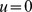.

Typically, we do not find convergence to one of the strict Nash equilibria (Fig. 3 b). In only 5% of the cases the trajectories converge to the pure ALLD equilibrium and in 18% of the cases the trajectories converge to the pure Grim equilibrium. However, in 77% of the cases we observe convergence to a mixed equilibrium of several strategies, dominated by Forgiver with a population share of 82.6% (Fig. 3 b). The other six strategies present in this cooperative alliance are Paradoxic Grateful (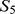; population share of 3.2%), Grateful (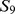; 5.6%), Suspicious ALLC (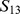; 3.8%), and ALLC (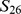; 0.3%), all of which have a sink-state C, and TFT (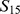; 4.1%) and WSLS (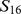; 0.4%), which are the remaining two dynamic strategies with the conditional cooperation element.

When increasing the benefit value to 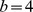 and 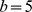, we observe convergence to a very similar alliance (Fig. 3 c and 3 d). For 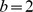, however, the ssC (sink-state C) strategies (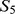, 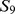, 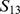, 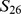) and WSLS are replaced by Grim and the mixed equilibrium is formed by Forgiver, TFT, and Grim (Fig. 3 a). Very rarely we observe convergence to a cooperative alliance led by Suspicious Forgiver (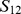; for short, sForgiver). It turns out that for some parameter values the Suspicious Forgiver alliance is an equilibrium (Fig. S5 and S7 in File S1).

**Figure 4:**
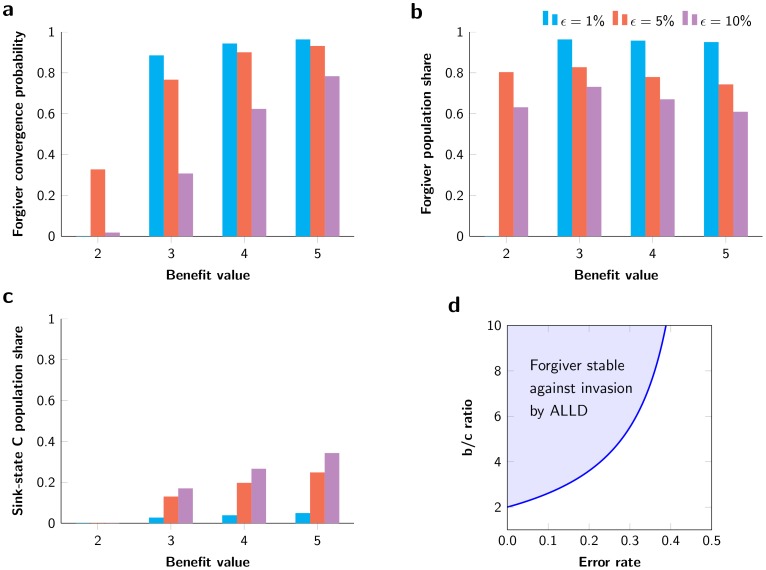
Robustness of results across various benefit values and error rates. **a**| Convergence probability to the Forgiver equilibrium of a uniform-random point in the 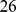-simplex. Note that for higher error rates (increasing noise-level), the probability to converge to the cooperative equilibrium is much lower. **b** | Population share of Forgiver (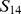) in the Forgiver equilibrium. Observe the relationship between the higher error rates and the lower population share of Forgiver. **c** | Population share of sink-state C strategies (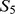, 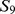, 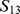, 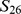) in the Forgiver equilibrium. Higher error rates lead to higher proportions of unconditional cooperators. **d** | In the infinitely repeated game, for all value pairs of 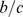 and 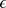 in the blue shaded area, ALLD cannot invade Forgiver since the average payoff of Forgiver playing against itself is higher than the average payoff of ALLD against Forgiver (see Inequality (1)).

From the 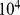 random initial frequencies, the four equilibria were reached in the proportions shown in Table 2 (using 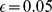 and 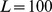; for other values of 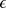 and 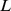 see Tables S9 and S10 in File S1). The mixed Forgiver equilibrium is the most commonly observed outcome. Note that in the case of 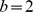 the mixed Forgiver equilibrium has a very different composition than in the cases of 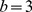, 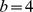, 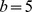. Changing the error rate, 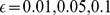 and the average number of rounds per game, 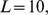 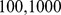, we find very similar behavior. Only the frequencies of the strategies within the mixed equilibria change marginally but not the general equilibrium composition (Fig. 4). Though, there is one exception. When the probability for multiple errors within an entire match becomes very low (e.g., 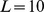 and 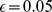 or 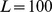 and 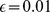) and 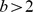, the payoff of ALLC against Grim can become higher than the payoff of Grim against itself. In other words, Grim can be invaded by ALLC. Hence, instead of the pure Grim equilibrium we observe a mixed equilibrium between Grim and ALLC (Fig. S4b–d in File S1).

**Table 2:** Equilibrium frequencies.

	ALLD	Grim	Forgiver	sForgiver
**b = 2**	15%	52%	33%	<1%
**b = 3**	5%	18%	77%	0%
**b = 4**	2%	7%	90%	1%
**b = 5**	1%	3%	93%	3%.

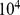
 uniformly distributed random points in the 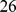-simplex. In the case of 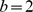, the mixed Forgiver equilibrium has a different composition than in the cases of 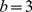, 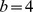, 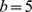. Parameter values: costs 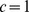, error rate 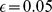, number of rounds per game 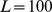. Proportions in which the four equilibria were reached from

We check the robustness of the observed equilibria by incorporating mutation to the replicator equation. We find that both the ALLD and the rare Suspicious Forgiver equilibrium are unstable. In the presence of mutation the evolutionary trajectories lead away from ALLD to Grim and from Suspicious Forgiver to Forgiver (see Fig. S6 and S7 in File S1). The Grim equilibrium and the Forgiver equilibrium remain stable. We note that this asymptotic stability is also due to the restricted strategy space. In [51] it has been shown that in the simultaneous Prisoner's Dilemma with an unrestricted strategy space, no strategy is robust against indirect invasions and hence, no evolutionarily stable strategy can exist.

Essential for the stability in our model is that Forgiver can resist invasion by ssD strategies (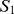, 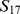, 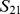, 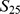), because Forgiver does better against itself than the ssD strategies do against Forgiver (Table 1). However, Forgiver can be invaded by ssC strategies and TFT. But, since TFT performs poorly against itself and ssC strategies are exploited by WSLS (Table 1), all these strategies can coexist in the Forgiver equilibrium. Stable alliances of cooperative strategies have also been found in the context of the Public Goods Game [52] and indirect reciprocity [53]. More detailed results and equilibrium analysis for a wide range of parameter values for 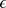 and 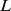 are provided in File S1 (Tables S1–S14 and Figures S2–S7).

In the limit of infinitely many rounds per game, we cancase of an infinitely repeated game, and we derive analytical results for the average payoff per round for the most relevant strategy pairs (Table 3; for the calculations see File S1: Section 2 and Fig. S8–S10). From these results we obtain that ALLD (or ssD strategies) cannot invade Forgiver if
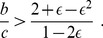
(1)This result holds for any error rate, 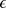, between 0 and 1/2 (Fig. 4 d).

**Table 3:** Analytical results in the infinitely alternating Prisoner's Dilemma.

	ALLD	Forgiver	ALLC
**ALLD**	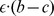	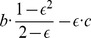	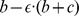
**Forgiver**	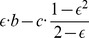	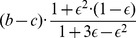	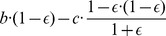
**ALLC**	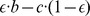	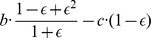	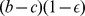

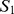
), Forgiver (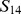), and ALLC (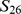) playing against each other. Derivations are provided in File S1 (section 2). Analytical results of the average payoff per round in the infinitely alternating Prisoner's Dilemma for ALLD (

## Discussion

Our results imply an indisputable strength of the strategy Forgiver in the alternating Prisoner's Dilemma in the presence of noise. For a wide range of parameter values, Forgiver is the dominating strategy of the cooperative equilibrium, having a population share of more than half in all investigated scenarios.

Essential for the success of a cooperative strategy in the presence of noise is how fast it can recover back to cooperation after a mistake, but at the same time, also avoid excessive exploitation by defectors. The conditional cooperation element is crucial for the triumph of Forgiver. Even though, also TFT and WSLS contain this element, which allows them to cooperate against cooperative strategies without getting excessively exploited by defectors, these strategies are not as successful as Forgiver, because of their inability to correct errors. Grim also possesses this conditional cooperation element. However, noise on the part of Grim's opponent will inevitably cause Grim to switch to always-defect. It is Grim's ability to conditionally cooperate for the first handful of turns that provides a competitive advantage over pure ALLD such that the strict Nash equilibrium ALLD can only rarely arise.

The other strategies appearing in the Forgiver equilibrium for the cases of 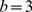, 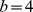, and 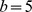 are Paradoxic Grateful (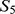), Grateful (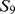), Suspicious ALLC (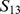), and ALLC (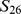). All of them are ssC strategies that, in the presence of noise, behave like ALLC after the first few moves. The strategy ALLC does very well in combination with Forgiver. Nevertheless, ALLC itself appears rarely. Perhaps because of Paradoxic Grateful, which defects against ALLC for many moves in the beginning, whereas Suspicious ALLC puts Paradoxic Grateful into its cooperating state immediately. One might ask why these ssC strategies do not occupy a larger population share in the cooperative equilibrium. The reason is the presence of exploitative strategies like WSLS which itself is a weak strategy in this domain. If only Forgiver was present, WSLS would be quickly driven to extinction; WSLS does worse against itself and Forgiver than Forgiver does against WSLS and itself (see Table 1). But WSLS remains in the Forgiver equilibrium because it exploits the ssC strategies. Interestingly, higher error rates increase the population share of unconditional cooperators (ssC strategies) in the cooperative equilibrium (Fig. 4c). Simultaneously, the higher error rates can decrease the probability to converge to the cooperative equilibrium dramatically and hence prevent the evolution of any cooperative behavior (Fig. 4a).

Grim and Forgiver are similar strategies, the difference being, in the face of a defection, Forgiver quickly returns to cooperation whereas Grim never returns. An interesting interpretation of the relationship is that Grim never forgives while Forgiver always does. Thus, the clash between Grim and Forgiver is actually a test of the viability of forgiveness under various conditions. On the one hand, the presence of noise makes forgiveness powerful and essential. On the other hand, if cooperation is not valuable enough, forgiveness can be exploited. Moreover, even when cooperation is valuable, but the population is ruled by exploiters, forgiveness is not a successful strategy. Given the right conditions, forgiveness makes cooperation possible in the face of both exploitation and noise.

These results demonstrate a game-theoretic foundation for forgiveness as a means of promoting cooperation. If cooperation is valuable enough, it can be worth forgiving others for past wrongs in order to gain future benefit. Forgiving incurs a short-term loss but ensures a greater long-term gain. Given all the (intentional or unintentional) misbehavior in the real world, forgiveness is essential for maintaining healthy, cooperative relationships.

## Methods

### Strategy space

We consider deterministic finite automata [41] (DFA) with one and two states. There are two one-state automata which encode the strategies always-defect (ALLD) and always-cooperate (ALLC). In total, there are 32 two-state automata encoding strategies in our game: two possible arrangements of states (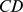, 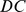) and 16 possible arrangements of transitions per arrangement of states. For 8 of these 32 automata, the second state is not reachable, making them indistinguishable from a one-state automata. Since we already added the one-state automata to our strategy space, these 8 can be ignored. The remaining 24 two-state automata encode distinct strategies in our game. Hence, in total we have 26 deterministic strategies in the alternating Prisoner's Dilemma (Fig. 1).

### Generation of the payoff matrix

In each round of the game a player can either cooperate or defect. Cooperation means paying a cost, 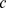, for the other player to receive a benefit, 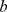. Defection means paying no cost and distributing no benefit. If 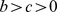 and we sum over two consecutive moves (equivalent to one round), the game is a Prisoner's Dilemma since the following inequality is satisfied: 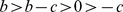. In other words, in a single round it is best to defect, but cooperation might be fruitful when playing over multiple rounds. Furthermore also 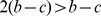 holds, and hence mutual cooperation results in a higher payoff than alternating between cooperation and defection.The second inequality ensures that sustained cooperation results in a higher payoff than alternation between cooperation and defection.

For each set of parameters (number of rounds 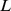, error rate 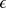, benefit value 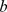, and costs 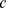), we generate a 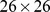 payoff matrix 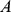 where each of the 26 distinct strategies is paired with each other. The entry 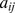 in the payoff matrix 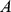 gives the payoff of strategy 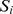 playing against strategy 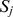. Based on the average of which strategy (player) goes first, we define the initial state distribution of both players as a row vector 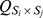. Since the players do not observe when they have made a mistake (i.e., the faulty player does not move to the corresponding state of the erroneous action which he has accidentally played), the state space consists of sixteen states namely 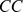, 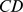, 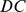, 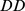, 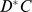, 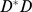, 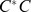, 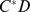, 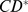, 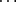 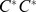. The star after a state indicates that the player accidentally played the opposite move as intended by her current state.

Each game consists of 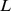 moves of both player. In each move, a player makes a mistake with probability 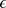 and thus implements the opposite move of what is specified by her strategy (automaton). We denote 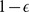 by 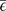. Although, the players do not observe their mistakes, the payoffs depend on the actual moves. This setting relates to imperceptive implementation errors [16], [18], [29], [45] (see section 3 in File S1 for a discussion on error types). The payoffs corresponding to their moves in the different states are given by the column vector 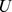.

Next, we define a 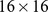 transition matrix 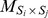 for each pair of strategies 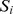, 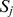. The entries of the transition matrix are given by the probabilities to move from eachone state of the sixteen states (defined above) to the next:
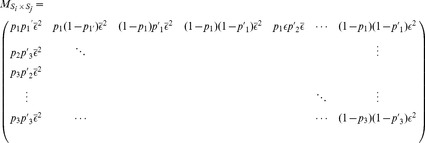
(2)where the quadruple [39] 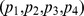 defines the probabilities of strategy 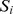 to cooperate in the observed states 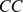, 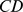, 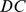, and 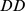 (errors remain undetected by the players). Respectively, the quadruple 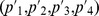 encodes the strategy 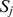. For example, 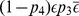 is the probability to move from state 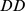 to state 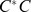. A deterministic strategy is represented as a quadruple where each 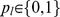.

Using the initial state distribution 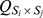, the transition matrix 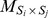, and the payoff vector 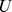, we calculate the payoff 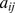 of strategy 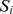 playing against strategy 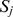 via a Markov Chain:
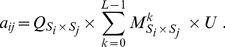
(3)Applying equation (3) to each pair of strategies, we obtain the entire payoff matrix 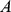 for a given set of parameter values. Although we use deterministic strategies, the presence of noise implies that the game that unfolds between any two strategies is described by a stochastic process. Payoff matrices for benefit values of 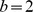, 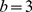, 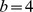, and 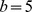, for error rates of 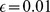, 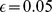, and 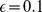, and for game length of 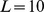, 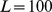, and 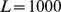 are provided in File S1 (Tables S1–S8).

#### Evolution of strategies

The strategy space spans a 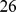-simplex which we explore via the replicator equation [48]–[50] with and without mutations. The frequency of strategy 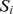 is given by 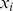. At any time 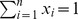 holds where 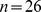 is the number of strategies. The average payoff (fitness) for strategy 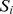 is given by
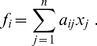
(4)The frequency of strategy 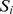 changes according to the differential equation
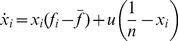
(5)where the average population payoff is 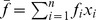 and 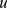 is the mutation rate. Mutations to each strategy are equally likely; for non-uniform mutation structures see [54]. Using the differential equation (5), defined on the 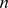-simplex (here, 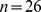), we study the evolutionary dynamics in the alternating Prisoner's Dilemma for many different initial conditions (i.e., random initial frequencies of the strategies). We generate a uniform-random point in the 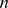-simplex by taking the negative logarithm of 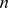 random numbers in 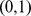, then normalizing these numbers such that they sum to 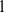, and using the normalized values as the initial frequencies of the 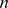 strategies [55].

#### Computer simulations

Our computer simulations are implemented in Python and split into three programs. The first program generates the 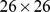 payoff matrix for each set of parameters. The second program simulates the deterministic selection dynamics starting from uniform-random points in the 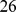-simplex. The third program performs statistical analysis on the results of the second program. The code is available at http://pub.ist.ac.at/~jreiter upon request [56].

## Supporting Information

File S1 **Detailed description of the model and the strategies; Simulation results and equilibrium analysis for a wide range of parameter values; Calculations for the infinitely-repeated game; Implementation of errors; Includes Tables S1–S14 and Figures S1–S10.**
(PDF)
