# Correction: Forgiver Triumphs in Alternating Prisoner's Dilemma

**DOI:** 10.1371/annotation/a3480abf-52e4-43f3-b45c-b2bac5e84812

**Published:** 2014-01-17

**Authors:** Benjamin M. Zagorsky, Johannes G. Reiter, Krishnendu Chatterjee, Martin A. Nowak

The Supporting Information was incorrectly omitted from the article. Please view the Supporting Information here: 
