# Supplementary material for: Correction: Forgiver Triumphs in Alternating Prisoner's Dilemma
Source: PLoS One. 2014 Jan 17;9(1):10.1371/annotation/a3480abf-52e4-43f3-b45c-b2bac5e84812. doi: 10.1371/annotation/a3480abf-52e4-43f3-b45c-b2bac5e84812 (PMC3897288; doi:10.1371/annotation/a3480abf-52e4-43f3-b45c-b2bac5e84812)
Supplement: Supplementary file 1 [file pone.a3480abf-52e4-43f3-b45c-b2bac5e84812.s001.pdf]

Supplementary Information for

Forgiver triumphs in alternating Prisoner's Dilemma

Benjamin M. Zagorsky[1], Johannes G. Reiter[2], Krishnendu Chatterjee[2], and Martin A. Nowak[1,3]

[1]Program for Evolutionary Dynamics, Harvard University, Cambridge 02138, USA
[2]IST Austria (Institute of Science and Technology Austria), Klosterneuburg 3400, Austria
[3]Department of Mathematics, Department of Organismic and Evolutionary Biology, Harvard University, Cambridge 02138, USA

We present additional results for the alternating Prisoner's Dilemma where each player can either cooperate or defect in response to the last move of the opponent. We recall that a cooperating player incurs a cost c resulting in a benefit b for the other player ($0 < c < b$). Hence, summing over two consecutive moves, if both players cooperate, each one receives a payoff of $b - c$; if both defect, they are left with nothing. If one player cooperates and the other player defects, the cooperator receives the lowest payoff of $-c$ and the defector receives the highest payoff of b. We obtain the following payoff matrix:

	**C**	**D**
**C**	$b - c,\ b - c$	$-c,\ b$
**D**	$b,\ -c$	$0,\ 0$.

One game consists of L moves of each player. In each round, a player makes a mistake with probability ϵ and thus implements the opposite move of what is specified by her strategy (automaton). The 26×26 payoff matrix where each of the 26 distinct strategies is paired with each other are calculated using equation (3) of the main paper. We show parts of the payoff matrices

with the most relevant strategies for different benefit values, error rates, and number of rounds in Tables S1, S2, S3, S4, S5, S6, S7, and S8.

Strategies. We consider 26 unique deterministic strategies implemented by one and two-state automata. Strategies $S_1 - S_{13}$ start in state D while strategies $S_{14} - S_{26}$ start in state C. Within these groups the strategies differ only in their transitions between the states. Some strategies have states without outgoing transitions, these strategies simplify to sink-state C (ssC) or sink-state D (ssD) strategies. Sink-state strategies always-cooperate or always-defect either from the beginning or after some condition is met. All remaining strategies are dynamic strategies and switch their state depending on the moves of their opponent (see Figure 1). A subset of these strategies (Forgiver, TFT, WSLS, and their suspicious counterparts) is especially interesting as they have the design element to stay in the cooperative state if the opponent has cooperated in the last round but switch to defection if the opponent has defected (see Figure S1). We call this element the conditional cooperation element.

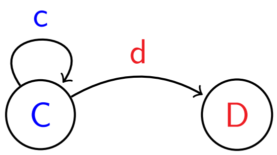

Figure S1: The conditional cooperation element. The success of the strategies Forgiver (S_{14}), TFT (S_{15}), WSLS (S_{16}), Grim (S_{17}), S_4, S_8, and S_{12} is largely due to the conditional cooperation element which allows them to benefit from mutual cooperation but also avoids excessive exploitation by defectors.

Here we list all considered strategies ordered by ascending indexes: S_1 (ALLD), S_2, S_3 (Suspicious Paradoxic), S_4 (Suspicious WSLS), S_5 (Paradoxic Grateful), S_6, S_7, S_8 (Suspicious TFT), S_9 (Grateful), S_{10} (Suspicious Alternator), S_{11}, S_{12} (Suspicious Forgiver), S_{13} (Suspicious ALLC), S_{14} (Forgiver), S_{15} (TFT), S_{16} (WSLS), S_{17} (Grim), S_{18}, S_{19}, S_{20} (Paradoxic), S_{21} (Paradoxic Grim), S_{22} (Alternator), S_{23}, S_{24}, S_{25} (Hopeful ALLD), S_{26} (ALLC).

	ALLD	pGrateful	Grateful	sForgiver	sALLC	Forgiver	TFT	WSLS	Grim	ALLC
**ALLD**	5.0	184.0	150.9	97.4	184.1	97.3	14.9	95.0	6.0	185.0
**pGrateful**	-84.5	86.1	94.0	85.9	103.5	85.8	85.3	-1.8	-82.2	112.1
**Grateful**	-67.9	94.2	85.9	86.6	95.0	86.5	82.1	14.4	-58.2	95.5
**sForgiver**	-41.2	98.0	97.8	87.1	98.7	87.0	79.6	82.4	-32.9	99.7
**sALLC**	-84.6	76.7	93.6	86.2	94.6	86.1	85.7	6.2	-65.8	95.5
**Forgiver**	-41.1	98.2	97.8	87.0	98.8	87.4	79.9	83.1	-26.9	99.3
**TFT**	0.1	98.4	94.0	90.5	99.0	90.9	52.2	90.1	10.2	99.5
**WSLS**	-40.0	141.9	133.8	84.2	138.7	84.8	79.4	53.3	-26.1	135.9
**Grim**	4.5	182.2	158.2	96.5	174.7	96.6	22.3	94.5	14.1	167.9
**ALLC**	-85.0	60.9	94.0	85.7	94.1	86.5	86.0	13.2	-50.9	95.0

Table S1: Excerpt of the payoff matrix with the most relevant strategies when the benefit value $b = 2$, the error rate $\epsilon = 5\%$, and the number of rounds in each game $L = 100$. There are two pure Nash equilibria in the full payoff matrix: ALLD (S_1) and Grim (S_{17}), both denoted in red.

	ALLD	pGrateful	Grateful	sForgiver	sALLC	Forgiver	TFT	WSLS	Grim	ALLC
**ALLD**	10.0	278.5	228.8	148.5	278.7	148.4	24.8	144.9	11.5	280.0
**pGrateful**	-79.5	172.1	188.0	175.8	198.0	175.7	174.9	44.4	-76.3	207.1
**Grateful**	-62.9	188.3	171.9	176.9	189.6	176.8	168.1	68.5	-44.2	190.5
**sForgiver**	-36.2	192.0	191.8	174.1	193.3	174.0	162.8	165.3	-22.7	194.7
**sALLC**	-79.6	162.3	187.6	176.5	189.1	176.4	175.8	56.6	-51.4	190.5
**Forgiver**	-36.1	192.2	191.8	174.1	193.3	174.8	163.5	166.9	-12.7	194.3
**TFT**	5.1	192.4	184.0	177.3	193.5	178.1	104.5	176.7	24.5	194.5
**WSLS**	-35.0	236.0	227.8	167.8	233.3	169.1	162.3	106.5	-12.0	230.9
**Grim**	9.5	276.2	244.2	149.8	269.3	152.0	40.5	148.8	28.1	262.9
**ALLC**	-80.0	138.8	188.5	176.0	188.7	177.2	176.6	67.3	-28.8	190.0

Table S2: Excerpt of the payoff matrix with the most relevant strategies when the benefit value $b = 3$, the error rate $\epsilon = 5\%$, and the number of rounds in each game $L = 100$. There are two pure Nash equilibria in the full payoff matrix: ALLD (S_1) and Grim (S_{17}), both denoted in red.

	ALLD	pGrateful	Grateful	sForgiver	sALLC	Forgiver	TFT	WSLS	Grim	ALLC
**ALLD**	15.0	373.0	306.7	199.7	373.2	199.5	34.7	194.9	17.0	375.0
**pGrateful**	-74.5	258.2	282.0	265.7	292.6	265.6	264.6	90.5	-70.4	302.1
**Grateful**	-57.9	282.4	257.8	267.3	284.1	267.0	254.2	122.7	-30.3	285.5
**sForgiver**	-31.2	285.9	285.9	261.2	287.8	261.1	246.1	248.3	-12.5	289.7
**sALLC**	-74.6	248.0	281.7	266.9	283.7	266.7	265.9	107.0	-37.1	285.5
**Forgiver**	-31.1	286.2	285.8	261.1	287.9	262.2	247.1	250.6	1.6	289.3
**TFT**	10.1	286.5	274.1	264.2	288.1	265.4	156.7	263.2	38.7	289.5
**WSLS**	-30.0	330.0	321.8	251.4	327.8	253.3	245.2	159.8	2.0	325.9
**Grim**	14.5	370.2	330.3	203.1	363.8	207.4	58.8	203.1	42.2	357.9
**ALLC**	-75.0	216.7	283.0	266.3	283.2	267.9	267.1	121.5	-6.7	285.0

Table S3: Excerpt of the payoff matrix with the most frequent strategies when the benefit value $b = 4$, the error rate $\epsilon = 5\%$, and the number of rounds in each game $L = 100$. There are two pure Nash equilibria in the full payoff matrix: ALLD (S_1) and Grim (S_{17}), both denoted in red.

	ALLD	pGrateful	Grateful	sForgiver	sALLC	Forgiver	TFT	WSLS	Grim	ALLC
**ALLD**	20.0	467.5	384.7	250.9	467.8	250.6	44.6	244.9	22.5	470.0
**pGrateful**	-69.5	344.2	376.1	355.6	387.1	355.5	354.2	136.6	-64.5	397.1
**Grateful**	-52.9	376.5	343.7	357.6	378.7	357.3	340.2	176.9	-16.3	380.5
**sForgiver**	-26.2	379.9	379.9	348.2	382.4	348.1	329.3	331.3	-2.3	384.7
**sALLC**	-69.6	333.6	375.7	357.2	378.2	357.1	356.0	157.4	-22.7	380.5
**Forgiver**	-26.1	380.3	379.8	348.1	382.4	349.6	330.6	334.3	15.8	384.3
**TFT**	15.1	380.5	364.1	351.0	382.6	352.6	209.0	349.7	53.0	384.5
**WSLS**	-25.0	424.0	415.8	335.0	422.4	337.6	328.2	213.0	16.1	420.9
**Grim**	19.5	464.2	416.3	256.4	458.4	262.8	77.0	257.4	56.3	452.9
**ALLC**	-70.0	294.7	377.5	356.6	377.8	358.7	357.6	175.6	15.3	380.0

Table S4: Excerpt of the payoff matrix with the most frequent strategies when the benefit value $b = 5$, the error rate $\epsilon = 5\%$, and the number of rounds in each game $L = 100$. There are two pure Nash equilibria in the full payoff matrix: ALLD (S_1) and Grim (S_{17}), both denoted in red.

	ALLD	pGrateful	Grateful	sForgiver	sALLC	Forgiver	TFT	WSLS	Grim	ALLC
**ALLD**	2.0	294.5	111.4	149.8	294.5	149.7	6.4	149.0	3.5	296.0
**pGrateful**	-95.5	135.9	195.8	193.2	227.9	193.2	193.1	-9.7	-92.6	259.5
**Grateful**	-34.5	196.6	113.1	194.7	197.5	194.6	153.1	114.9	28.3	198.5
**sForgiver**	-47.3	196.9	196.5	193.3	198.0	193.3	190.5	190.5	7.7	199.4
**sALLC**	-95.5	104.4	195.5	194.2	197.0	194.2	194.1	52.7	-1.4	198.5
**Forgiver**	-47.2	196.9	196.5	193.3	198.0	194.2	191.4	192.4	59.5	199.0
**TFT**	0.5	197.0	155.3	194.2	198.0	195.2	124.1	195.1	86.6	199.0
**WSLS**	-47.0	264.6	223.0	190.7	245.1	192.5	191.4	133.9	59.3	226.2
**Grim**	1.5	292.2	171.7	159.6	263.2	170.1	88.8	169.6	86.9	234.5
**ALLC**	-96.0	13.4	196.5	193.7	196.5	195.1	195.1	113.5	88.6	198.0

Table S5: Excerpt of the payoff matrix with the most relevant strategies when the benefit value $b = 3$, the error rate $\epsilon = 1\%$, and the number of rounds in each game $L = 100$. There is only one pure Nash equilibria in the full payoff matrix: ALLD (S_1), denoted in red. The pure Grim equilibrium disappears when the probability for errors within an entire match becomes significantly smaller than one (i.e., $1 - (1 - \epsilon)^{2L} \ll 1$) and $b > 2$.

	ALLD	pGrateful	Grateful	sForgiver	sALLC	Forgiver	TFT	WSLS	Grim	ALLC
**ALLD**	20.0	258.5	238.0	146.5	258.8	146.2	45.1	139.9	21.5	260.0
**pGrateful**	-59.5	172.0	178.0	157.4	183.2	157.1	154.8	56.5	-56.6	187.3
**Grateful**	-52.7	178.5	171.7	158.2	179.7	157.9	152.7	65.9	-44.0	180.5
**sForgiver**	-22.2	184.8	184.9	156.1	186.3	156.0	138.9	142.1	-15.0	187.6
**sALLC**	-59.6	167.3	177.7	157.8	179.2	157.7	155.6	61.3	-46.5	180.4
**Forgiver**	-22.1	185.2	184.8	156.0	186.4	156.5	139.3	143.2	-11.2	187.2
**TFT**	11.6	185.9	182.6	160.6	187.1	161.2	102.0	156.5	21.0	188.0
**WSLS**	-20.0	218.7	215.4	149.3	218.5	150.1	136.3	102.9	-9.4	218.3
**Grim**	19.5	256.4	244.0	146.4	254.4	147.4	51.0	141.4	28.3	252.7
**ALLC**	-60.0	158.0	178.5	157.3	178.8	158.3	156.1	65.1	-38.0	180.0

Table S6: Excerpt of the payoff matrix with the most relevant strategies when the benefit value $b = 3$, the error rate $\epsilon = 10\%$, and the number of rounds in each game $L = 100$. There are two pure Nash equilibria in the full payoff matrix: ALLD (S_1) and Grim (S_{17}), both denoted in red.

	ALLD	pGrateful	Grateful	sForgiver	sALLC	Forgiver	TFT	WSLS	Grim	ALLC
**ALLD**	1.0	26.5	7.3	15.0	26.6	14.7	3.6	14.4	2.5	28.0
**pGrateful**	-7.5	11.7	16.7	16.3	21.4	16.2	16.1	-0.6	-5.1	25.9
**Grateful**	-1.1	18.1	7.3	17.5	18.6	17.3	12.4	14.1	6.5	19.5
**sForgiver**	-3.7	17.1	17.0	16.9	18.4	16.8	15.9	15.4	4.8	19.8
**sALLC**	-7.6	8.1	16.6	17.1	18.1	17.0	17.0	6.7	3.8	19.4
**Forgiver**	-3.6	17.3	16.9	16.8	18.5	17.6	16.6	17.0	11.2	19.4
**TFT**	0.1	17.4	12.8	17.0	18.5	17.9	14.0	17.8	12.6	19.4
**WSLS**	-3.5	22.9	17.9	15.8	21.9	17.1	16.5	14.9	11.2	20.8
**Grim**	0.5	24.5	13.5	15.7	22.9	17.1	13.1	17.0	12.7	21.1
**ALLC**	-8.0	-1.7	17.5	16.6	17.6	17.8	17.7	13.6	12.7	19.0

Table S7: Excerpt of the payoff matrix with the most relevant strategies when the benefit value $b = 3$, the error rate $\epsilon = 5\%$, and the number of rounds in each game $L = 10$. There is only one pure Nash equilibria in the full payoff matrix: ALLD (S_1), denoted in red. The pure Grim equilibrium disappears when the probability for errors within an entire match becomes significantly smaller than one (i.e., $1 - (1-\epsilon)^{2L} \ll 1$) and $b > 2$.

	ALLD	pGrateful	Grateful	sForgiver	sALLC	Forgiver	TFT	WSLS	Grim	ALLC
**ALLD**	100.0	2798.5	2748.5	1484.7	2798.7	1484.5	236.3	1449.9	101.5	2800.0
**pGrateful**	-799.5	1882.0	1898.0	1770.0	1908.1	1769.9	1763.4	539.4	-796.3	1917.2
**Grateful**	-782.8	1898.3	1881.9	1771.2	1899.6	1771.1	1756.6	563.5	-764.0	1900.5
**sForgiver**	-361.6	1940.6	1940.4	1746.5	1941.9	1746.4	1631.9	1664.2	-348.1	1943.2
**sALLC**	-799.6	1872.2	1897.6	1770.8	1899.1	1770.7	1764.3	551.6	-771.3	1900.5
**Forgiver**	-361.5	1940.8	1940.3	1746.4	1941.9	1747.1	1632.5	1665.7	-338.1	1942.8
**TFT**	54.6	1942.9	1934.5	1780.3	1944.0	1781.1	1004.5	1765.6	74.0	1945.0
**WSLS**	-350.0	2351.0	2342.8	1687.2	2348.3	1688.5	1620.0	1006.5	-327.0	2345.9
**Grim**	99.5	2796.2	2764.0	1485.9	2789.2	1488.2	252.0	1453.8	118.1	2782.8
**ALLC**	-800.0	1848.5	1898.5	1770.3	1898.7	1771.5	1765.1	562.3	-748.5	1900.0

Table S8: Excerpt of the payoff matrix with the most relevant strategies when the benefit value $b = 3$, the error rate $\epsilon = 5\%$, and the number of rounds in each game $L = 100$. There are two pure Nash equilibria in the full payoff matrix: ALLD (S_1) and Grim (S_{17}), both denoted in red.

1 Equilibrium analysis

We show the convergence probabilities to all observed equilibria for various benefit values, error rates, and average numbers of rounds per game in Tables S9 and S10. Furthermore, we show the strategy frequencies in the Forgiver equilibrium for various settings in Tables S11, S12, and S13.

In Figures S2, S3, S4, and S5 we show convergence to all types of equilibria from randomly chosen starting points (Forgiver equilibrium is shown in the main text). The ALLD and the Suspicious Forgiver equilibrium can become unstable and diverge to other equilibria in the presence of mutation. When the mutation rate u and the benefit value b are sufficiently large, we observe that starting from pure ALLD ($x_1 = 1$) the evolutionary trajectories converge to the Grim equilibrium (Figure S6); starting from the observed Suspicious Forgiver equilibrium the trajectories converge to the Forgiver equilibrium (Figure S7).

Very rarely, the evolutionary trajectories from a random starting point in the 26-simplex converge to a Forgiver dominated saddle point instead of the precise Forgiver equilibrium. What happens is that one of the required strategies of the Forgiver equilibrium goes extinct during the convergence process due to the numerical inaccuracy of discrete computer simulations. These trajectories do not converge to the precise Forgiver equilibrium (since one of the required strategies is missing) and hence converge to a very similar but unstable saddle point. After converging to such a saddle point we add once a small deviation to all strategy frequencies (i.e., a uniformly distributed random number in $[0, 10^{-5})$ is added to all x_i) which leads to a subsequent convergence to the precise Forgiver equilibrium as usually observed.

For specific parameter settings, we sometimes observe convergence to a cooperative equilibrium dominated by Suspicious Forgiver (S_{12}; sForgiver) instead of Forgiver. This is rather unsurprising since the long-term behavior of Forgiver and its suspicious counterpart is very similar. Because for some population compositions and some parameter settings, sForgiver has a higher fitness than Forgiver, sForgiver can form a mixed equilibrium with several other strategies (Table S2; Figure S5).

When the probability for errors within an entire match becomes significantly smaller than one (i.e., $1 - (1 - \epsilon)^{2L} \ll 1$) and cooperation is valuable enough $b > 2$, the pure Grim equilibrium disappears (see Tables S5 and S7). In this case, Grim and ALLC can form a stable mixed equilibrium (Figure S4).

In the case of $b = 2$, the structure of the mixed Forgiver equilibrium is very different to cases where $b > 2$. When $b = 2$, Grim is a stable member of the Forgiver equilibrium and drives the ssC (sink-state C) strategies and WSLS to extinction. Forgiver has the potential weakness that against always-defect (which Grim eventually does), it alternates between cooperation and

defection and gets suckered every other turn. In the case of $b = 3$, this is a dispensable weakness because exploitation yields an average payoff of 1.5 against Forgiver, while cooperation yields an average payoff of 2. However, when $b = 2$, both defecting and cooperating with Forgiver yield an average payoff of 1. Thus, Grim, by exploiting Forgiver, can do as well against Forgiver as Forgiver does against itself and they co-exist in equilibrium (the stability of this coexistence is entirely due to TFT). Following the above reasoning, it is obvious why for $b < 2$, exploiting Forgiver is more lucrative than cooperating with it, and the Forgiver equilibrium can not exist. When $b < 2$, the strategy space is dominated by Grim or ALLD.

In Table S14 we show the level of cooperation in the mixed Forgiver equilibrium accross different error rates and average numbers of rounds. We calculate the fraction of the maximal achievable cooperation by dividing the payoff of the strategies present in the Forgiver equilibrium by the fitness of ALLC playing against itself. In all investigated scenarios the mixed Forgiver equilibrium ensures a very high level of cooperation.

**Benefit value**		**2**			**3**			**4**			**5**	
**Error rate**	**0.01**	**0.05**	**0.1**	**0.01**	**0.05**	**0.1**	**0.01**	**0.05**	**0.1**	**0.01**	**0.05**	**0.1**
**ALLD**	<0.01	0.16	0.43	<0.01	0.05	0.22	0.0	0.02	0.11	0.0	0.02	0.07
**Grim**	>0.99	0.53	0.55	-	0.19	0.47	-	0.07	0.26	-	0.03	0.15
**Grim/ALLC**	-	-	-	0.13	-	-	0.06	-	-	0.03	-	-
**Forgiver**	0.0	0.31	0.02	0.86	0.75	0.3	0.93	0.9	0.62	0.97	0.93	0.78
**sForgiver**	0.0	<0.01	<0.01	<0.01	0.0	0.02	0.01	0.01	0.0	0.01	0.02	<0.01

Table S9: Convergence probabilities to the ALLD, Grim, Grim/ALLC, Forgiver, and Suspicious Forgiver equilibrium from 10^4 random starting points when the number of rounds per game game is $L = 100$, the mutation rate is $u = 0$, and $c = 1$.

**Benefit value**		**2**			**3**			**4**			**5**	
**Rounds per game**	**10**	**100**	**1000**	**10**	**100**	**1000**	**10**	**100**	**1000**	**10**	**100**	**1000**
**ALLD**	0.21	0.16	0.16	0.05	0.05	0.04	0.01	0.02	0.02	0.01	0.01	0.01
**Grim**	0.79	0.53	0.53	-	0.19	0.2	-	0.07	0.07	-	0.03	0.03
**Grim/ALLC**	-	-	-	0.45	-	-	0.25	-	-	0.15	-	-
**Forgiver**	0.0	0.31	0.31	0.5	0.76	0.75	0.74	0.9	0.91	0.84	0.93	0.96
**sForgiver**	0.0	<0.01	<0.01	<0.01	0.0	0.0	<0.01	0.01	0.0	0.01	0.02	0.0

Table S10: Convergence probabilities to the ALLD, Grim, Grim/ALLC, Forgiver, and Suspicious Forgiver equilibrium from 10^4 random starting points when the error rate is $\epsilon = 0.05$, the mutation rate is $u = 0$, and $c = 1$.

	pGrateful	Grateful	sALLC	Forgiver	TFT	WSLS	Grim	ALLC
$b=2$	-	-	-	-	-	-	-	-
$b=3$	0.01	0.0	0.0	0.74	0.085	0.0	0.0	0.166
$b=4$	0.015	0.0	0.0	0.709	0.05	0.0	0.0	0.226
$b=5$	0.017	0.0	0.0	0.671	0.034	0.0	0.0	0.278

Table S11: Frequencies of the the most frequent strategies in the Forgiver equilibrium when the error rate is $\epsilon = 5\%$, the mutation probability is $u = 0$, and the number of rounds in each game is $L = 10$.

	pGrateful	Grateful	sALLC	Forgiver	TFT	WSLS	Grim	ALLC
$b=2$	0.0	0.0	0.0	0.802	0.157	0.0	0.041	0.0
$b=3$	0.032	0.056	0.038	0.826	0.041	0.004	0.0	0.003
$b=4$	0.054	0.085	0.056	0.778	0.018	0.009	0.0	0.0
$b=5$	0.081	0.117	0.049	0.742	0.005	0.007	0.0	0.0

Table S12: Frequencies of the the most frequent strategies in the Forgiver equilibrium when the error rate is $\epsilon = 5\%$, the mutation probability is $u = 0$, and the number of rounds in each game is $L = 100$.

	pGrateful	Grateful	sALLC	Forgiver	TFT	WSLS	Grim	ALLC
$b=2$	0.0	0.0	0.0	0.813	0.15	0.0	0.037	0.0
$b=3$	0.037	0.07	0.02	0.796	0.045	0.016	0.0	0.016
$b=4$	0.053	0.09	0.08	0.725	0.027	0.024	0.0	0.0
$b=5$	0.083	0.126	0.081	0.664	0.018	0.027	0.0	0.0

Table S13: Frequencies of the the most frequent strategies in the Forgiver equilibrium when the error rate is $\epsilon = 5\%$, the mutation probability is $u = 0$, and the number of rounds in each game is $L = 1000$.

	$L=10$	$L=100$	$L=1000$
$\epsilon = 0.01$	0.985	0.981	0.982
$\epsilon = 0.05$	0.936	0.929	0.931
$\epsilon = 0.1$	0.897	0.888	0.889

Table S14: Fraction of maximal achievable cooperation in the Forgiver equilibrium when the benefit value is $b = 3$ and the mutation rate is $u = 0$. This fraction is calculated by dividing the payoff of the strategies present in the Forgiver equilibrium by the fitness of ALLC playing against itself.

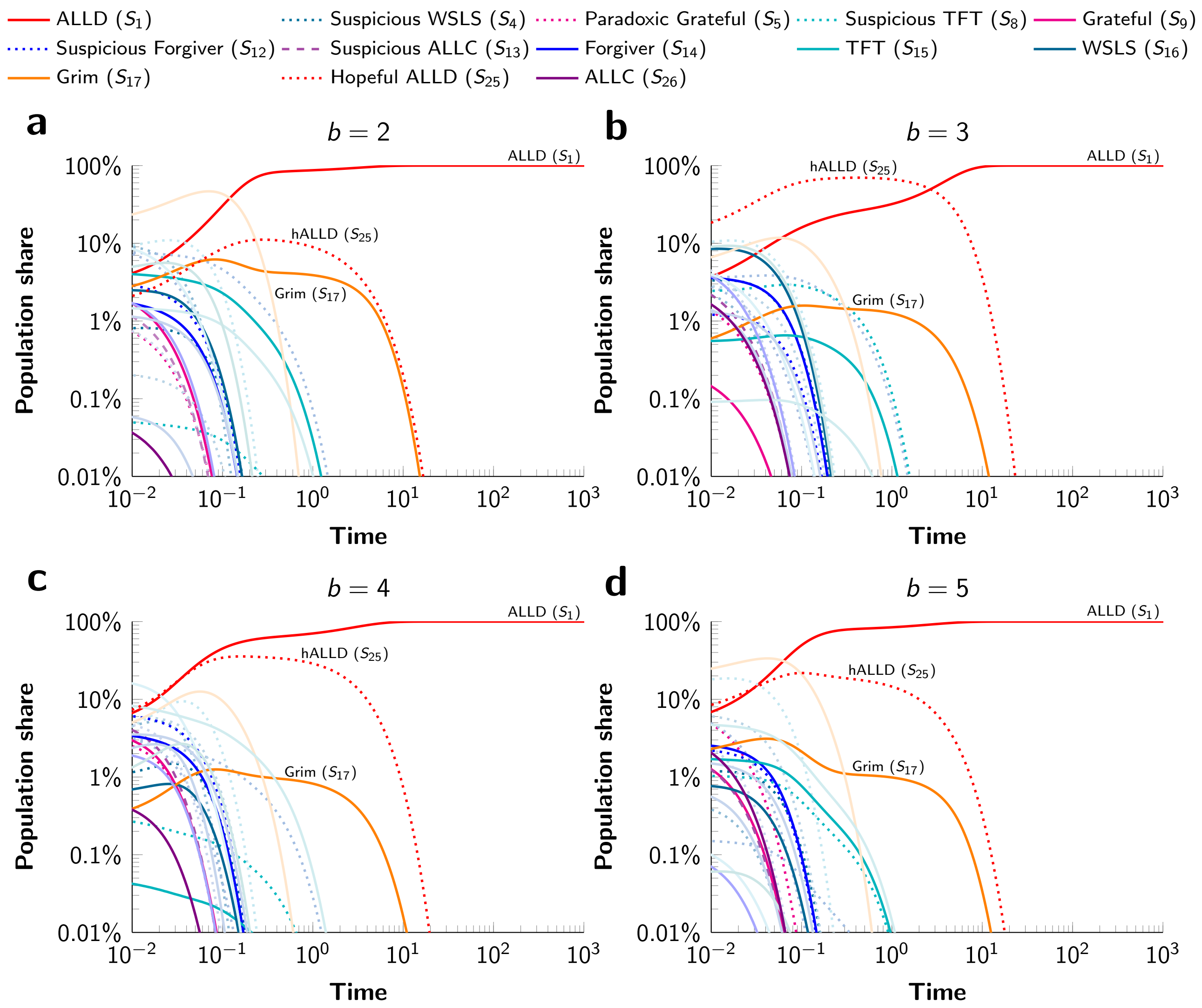

Figure S2: The evolution of the ALLD equilibrium in the alternating Prisoner's Dilemma. In all panels, the simulations start from a randomly chosen point in the 26-simplex. The error rate ϵ is set to 5%, the number of rounds per game is $L = 100$, and the mutation rate is $u = 0$.

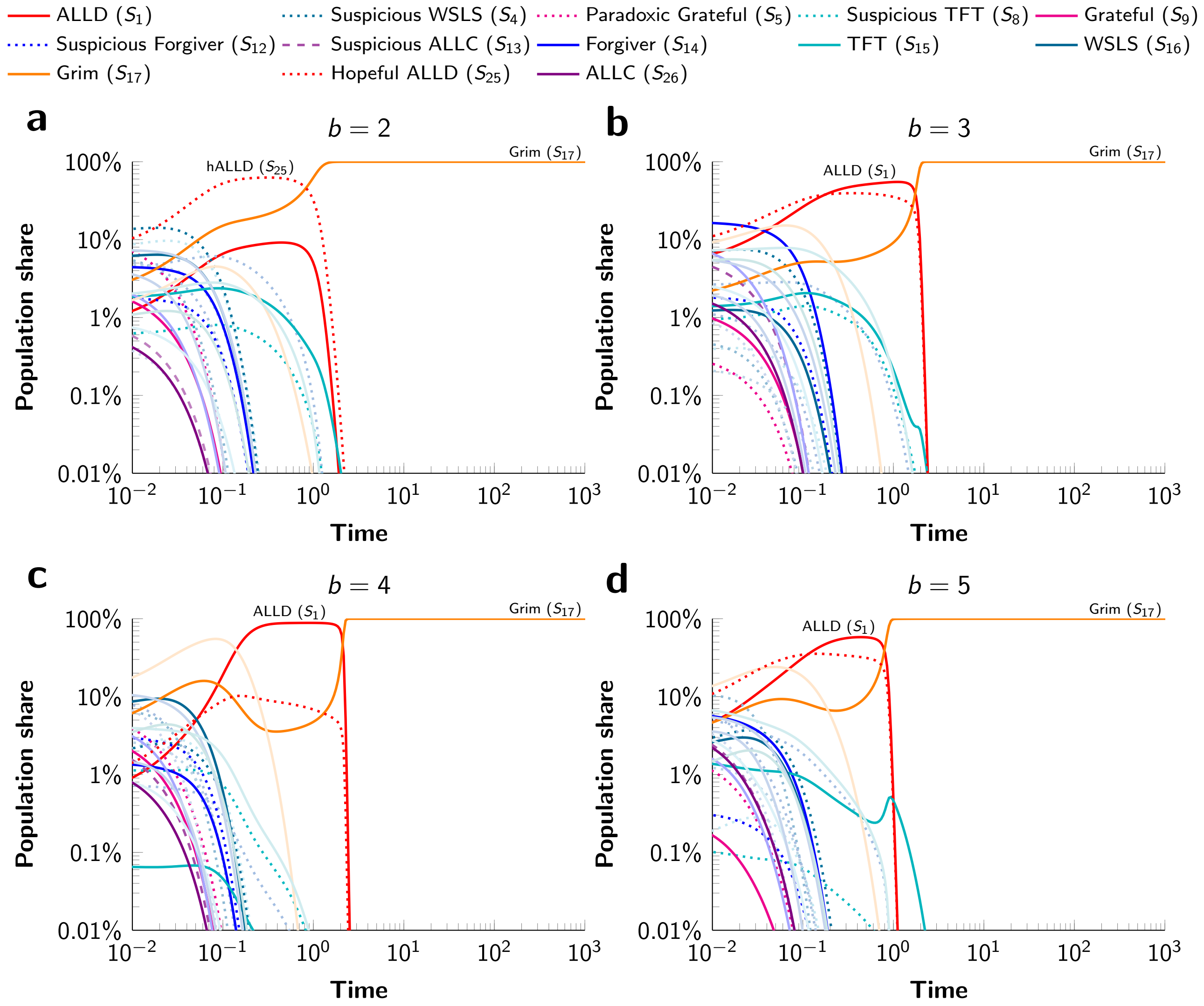

Figure S3: The evolution of the pure Grim equilibrium in the alternating Prisoner's Dilemma. In all panels, the simulations start from a randomly chosen point in the 26-simplex. The error rate ϵ is set to 5%, the number of rounds per game is $L = 100$, and the mutation rate is $u = 0$.

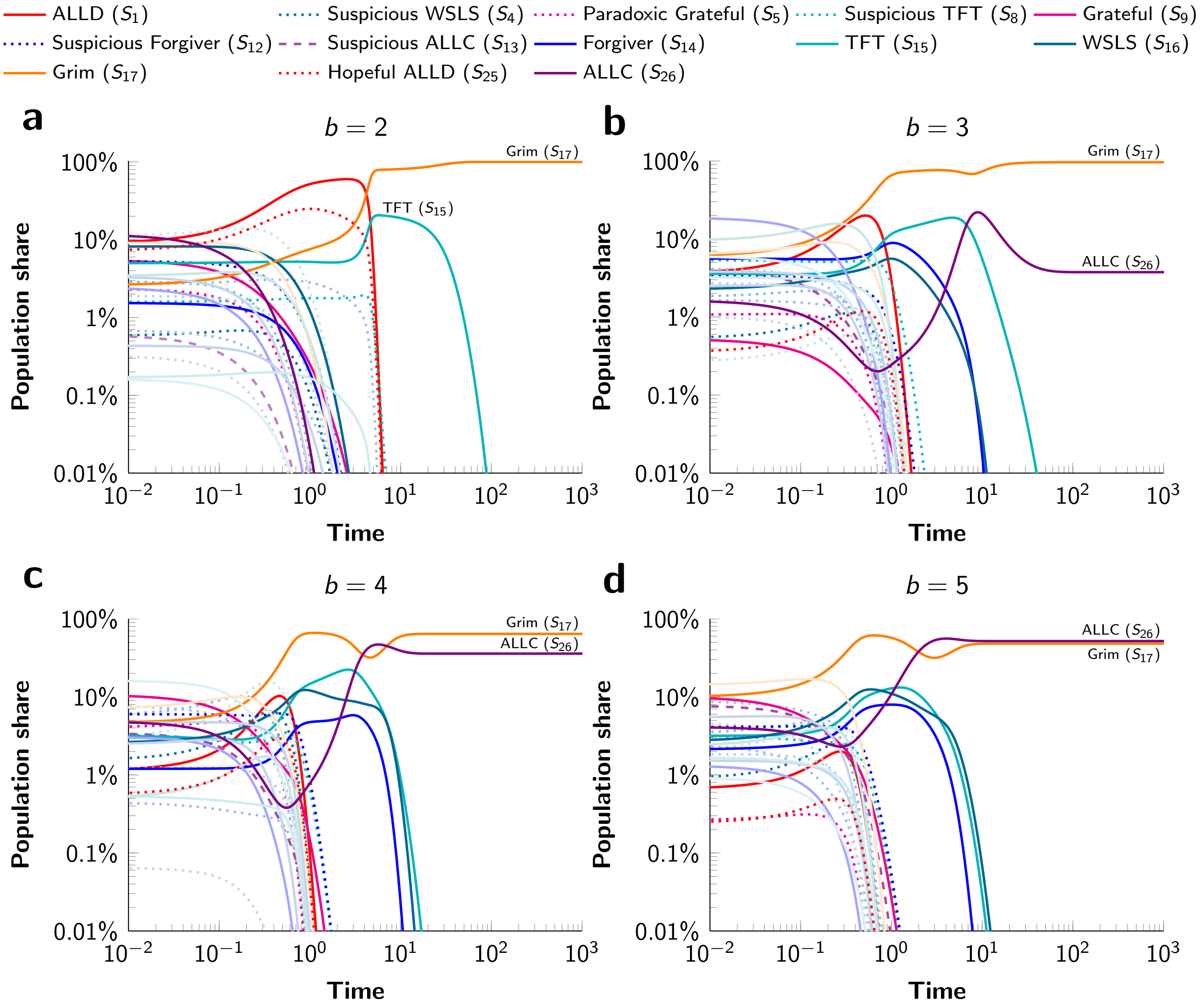

Figure S4: The evolution of the (mixed) Grim equilibrium in the alternating Prisoner's Dilemma when errors are rare. When the probability for errors within an entire match becomes significantly smaller than one (i.e., $1-(1-\epsilon)^{2L} \ll 1$) and $b > 2$, the pure Grim equilibrium turns into a mixed equilibrium with ALLC. In all panels, the simulations start from a randomly chosen point in the 26-simplex. The error rate ϵ is set to 5%, the number of rounds per game is $L = 10$, and the mutation rate is $u = 0$.

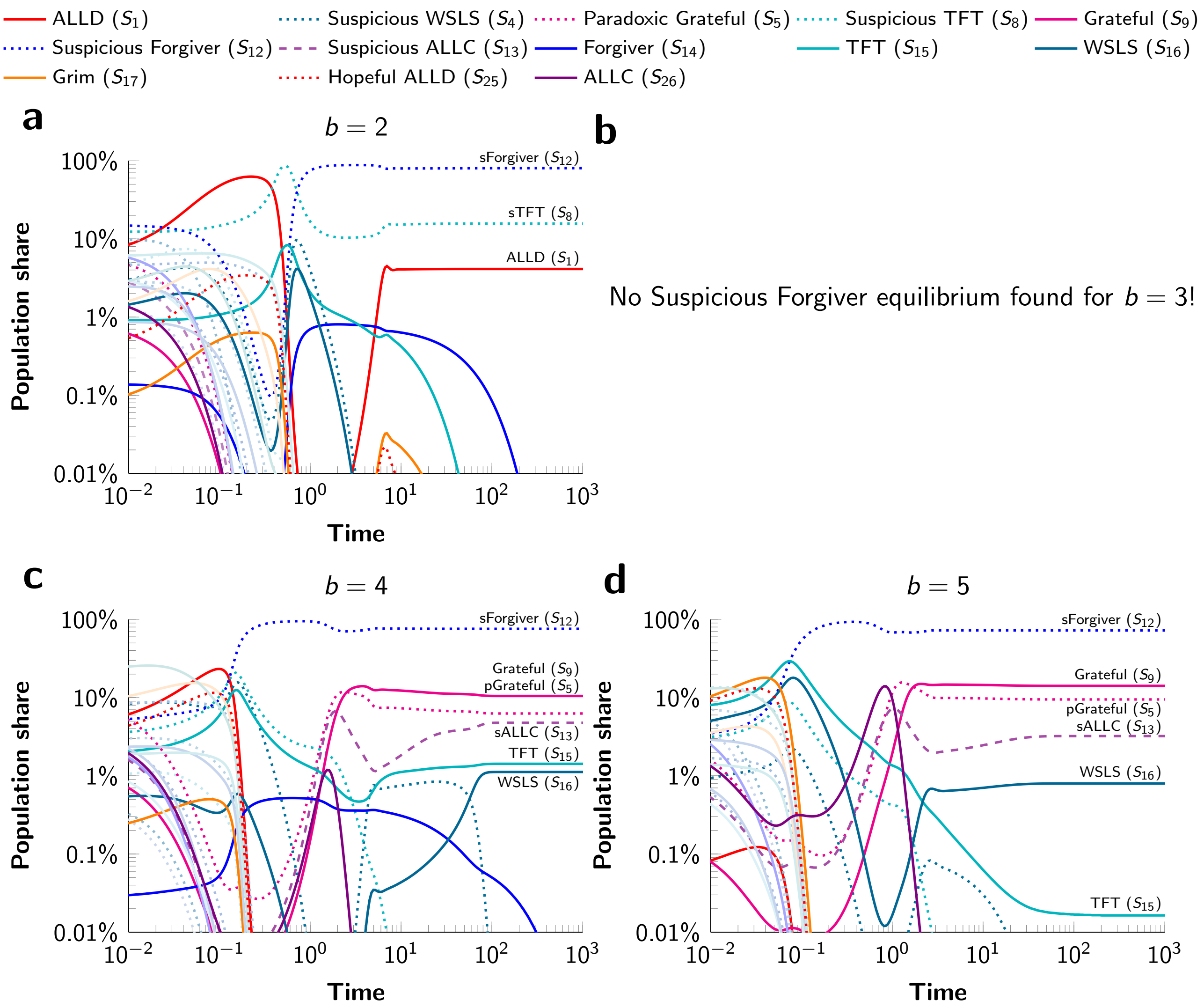

Figure S5: The evolution of the mixed Suspicious Forgiver equilibrium in the alternating Prisoner's Dilemma. In all panels, the simulations start from a randomly chosen point in the 26-simplex. The error rate ϵ is set to 5%, the number of rounds per game is $L = 100$, and the mutation rate is $u = 0$.

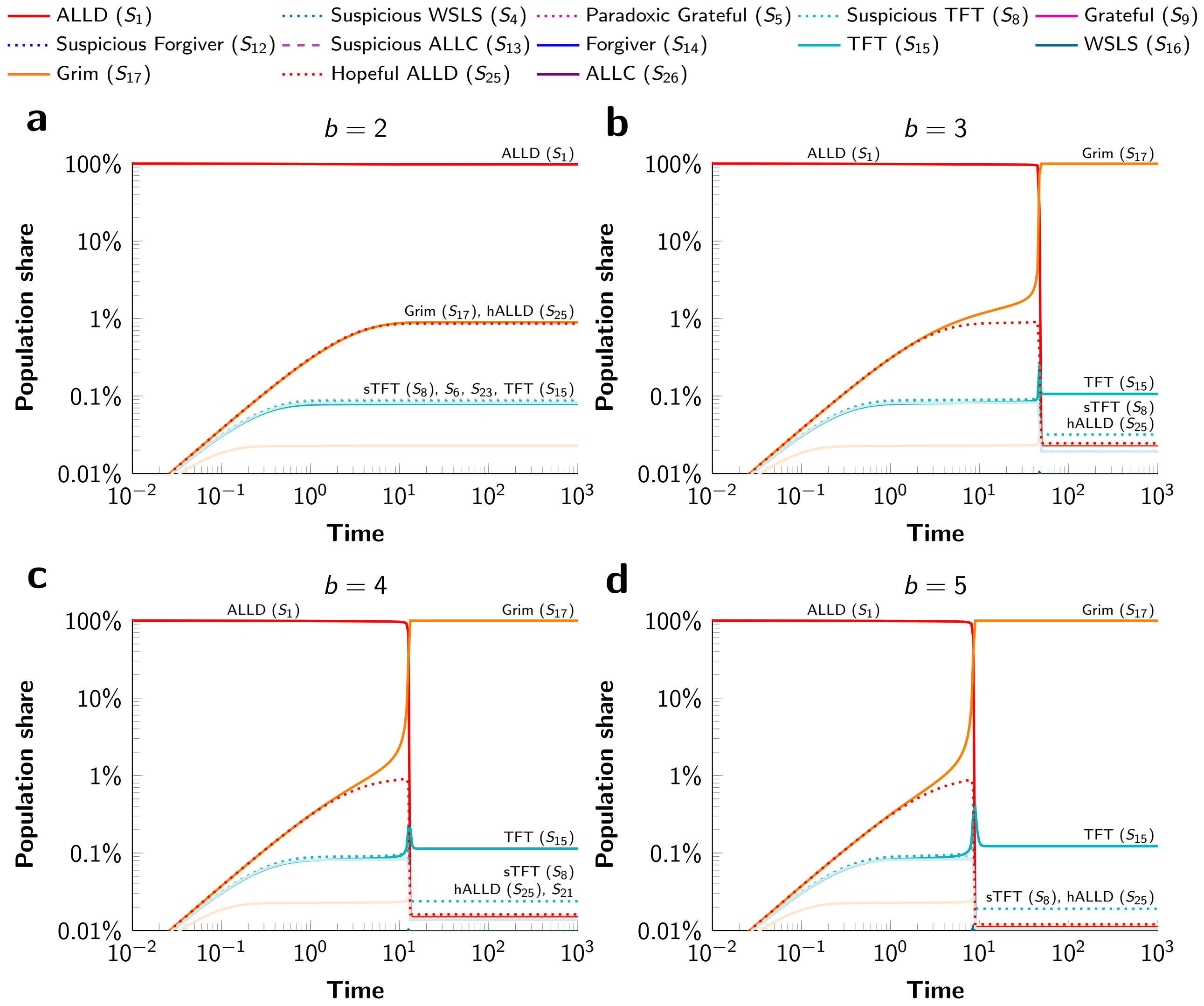

Figure S6: The ALLD equilibrium becomes unstable for high mutation rates. High mutation rates can lead to the evolution of Grim due to its higher payoff against cooperative strategies. If the mutation rate and the benefit value are sufficiently high, Grim outperforms ALLD and the simulation dynamics converge to a stable Grim equilibrium (panels b, c, and d). For smaller mutation rates or benefit values Grim cannot invade ALLD (panel a). In all panels, the simulations start from pure ALLD ($x_1 = 1$). The error rate ϵ is set to 5%, the number of rounds per game is $L = 100$, and the mutation rate is $u = 10\%$.

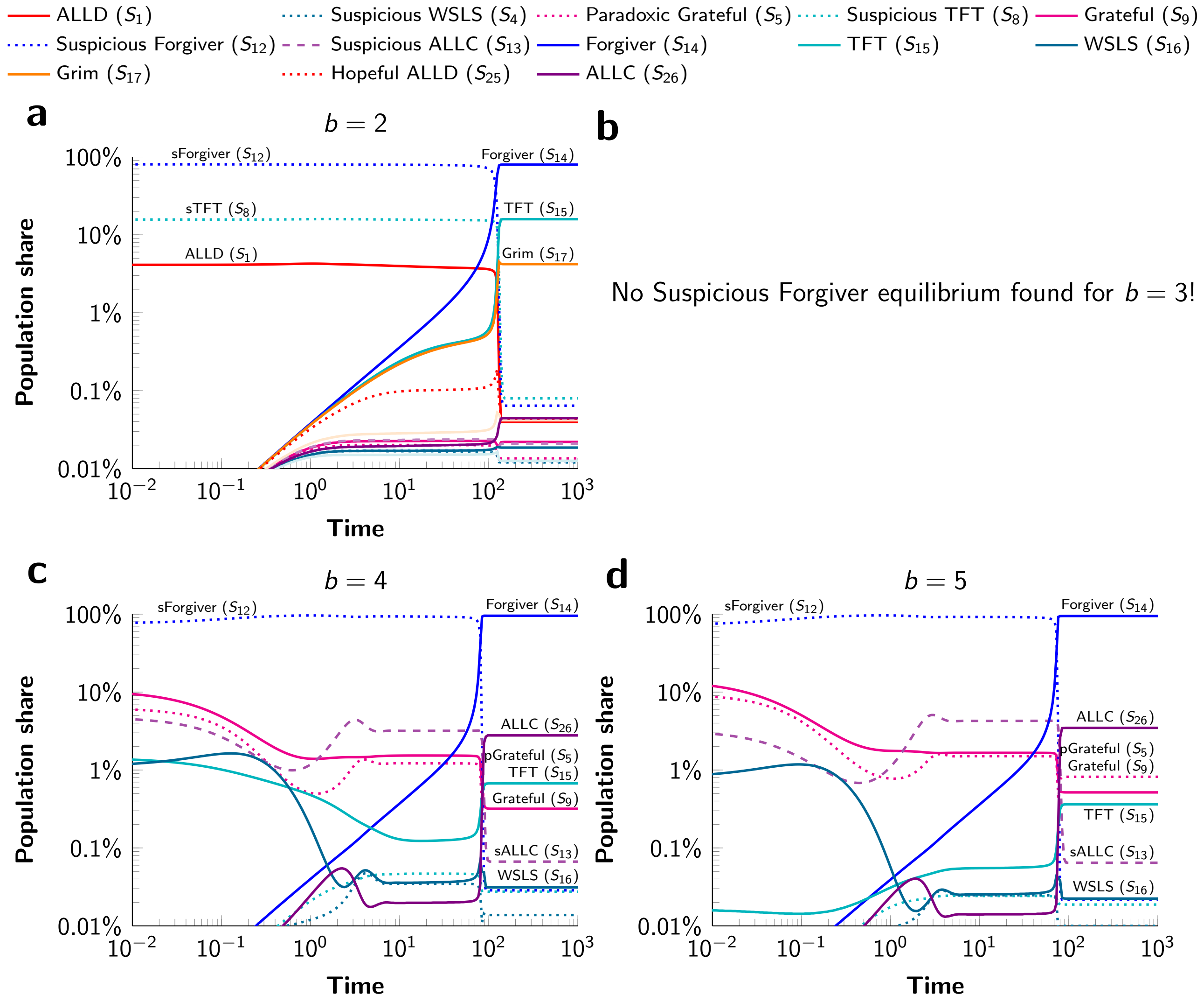

Figure S7: The Suspicious Forgiver equilibrium is unstable. In all panels, the simulations start from the mixed Suspicious Forgiver equilibrium. Small mutation rates can lead to the evolution of Forgiver due to its slightly higher payoff against cooperative strategies. If the mutation rate is sufficiently high, the simulation dynamics converge to the stable mixed Forgiver equilibrium. The error rate ϵ is set to 5%, the number of rounds per game is $L = 100$, and the mutation rate is $u = 1\%$.

2 Infinitely alternating Prisoner's Dilemma

We derive analytical results for the most relevant pairs of strategies playing the infinitely alternating Prisoner's Dilemma.

2.1 ALLD vs. ALLD

The average payoff per round of ALLD (S_1) playing against ALLD is:

$$R_{S_1 \times S_1} = 1 \cdot (1-\epsilon)^2 + \frac{b+1}{2} \cdot 2\epsilon \cdot (1-\epsilon) + b \cdot \epsilon^2 = 1 + \epsilon \cdot (b-1) \ . \tag{S1}$$

2.2 ALLD vs. ALLC

The average payoff per round of ALLD (S_1) playing against ALLC (S_{26}) is:

$$R_{S_1 \times S_{26}} = (b+1)(1-\epsilon)^2 + b \cdot \epsilon \cdot (1-\epsilon) + 1 \cdot (1-\epsilon) \cdot \epsilon + 0 \cdot \epsilon^2 = b + 1 - \epsilon \cdot (b+1) \ . \tag{S2}$$

The expected payoff per round of ALLC playing against ALLD is:

$$R_{S_{26} \times S_1} = 0 \cdot (1-\epsilon)^2 + 1 \cdot \epsilon \cdot (1-\epsilon) + b \cdot (1-\epsilon) \cdot \epsilon + (b+1) \cdot \epsilon^2 = \epsilon \cdot (b+1) \ . \tag{S3}$$

2.3 ALLC vs. ALLC

The average payoff per round of ALLC (S_{26}) playing against ALLC is:

$$R_{S_{26} \times S_{26}} = b \cdot (1-\epsilon)^2 + \frac{b+1}{2} \cdot 2\epsilon \cdot (1-\epsilon) + 1\epsilon^2 = b - \epsilon \cdot (b-1) \ . \tag{S4}$$

2.4 Forgiver vs. ALLD

In contrast to ALLD (S_1) and ALLC (S_{26}), the strategy Forgiver (S_{14}) is described by a two-state automaton. We calculate the frequency of each state by taking the product of the two automata encoding the strategies Forgiver and ALLD (Figure S8). In this new automaton we calculate the frequencies y_1 and y_2 of state (C, D) and (D, D), respectively. Since $y_2 = y_1(1-\epsilon)$ and $y_1 + y_2 = 1$ we obtain $y_1 = 1/(2-\epsilon)$ and $y_2 = (1-\epsilon)/(2-\epsilon)$. Based on these frequencies, we can take the results for ALLC vs. ALLD and ALLD vs. ALLD and compute the average payoff

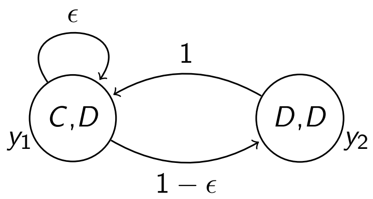

Figure S8: Product of the automata encoding Forgiver (S14) and ALLD (S1).

per round for Forgiver playing against ALLD as:

$$R_{S_{14}\times S_1} = \frac{1}{2-\epsilon}\cdot R_{S_{26}\times S_1} + \frac{1-\epsilon}{2-\epsilon}\cdot R_{S_1\times S_1} = \frac{1+\epsilon}{2-\epsilon} + \epsilon\cdot(b-1)\ . \tag{S5}$$

Similarly, the average payoff per round for ALLD vs. Forgiver is:

$$R_{S_1\times S_{14}} = \frac{1}{2-\epsilon}\cdot R_{S_1\times S_{26}} + \frac{1-\epsilon}{2-\epsilon}\cdot R_{S_1\times S_1} = \frac{1-\epsilon}{2-\epsilon}\left[2+b+\epsilon\cdot(b-1)\right]\ . \tag{S6}$$

2.5 Forgiver vs. ALLC

Again as above, we calculate the frequencies of each state in the automaton obtained by multiplying Forgiver and ALLC (Figure S9). Since $y_2 = \epsilon y_1$ and $y_1 + y_2 = 1$ we obtain $y_1 = 1/(1+\epsilon)$

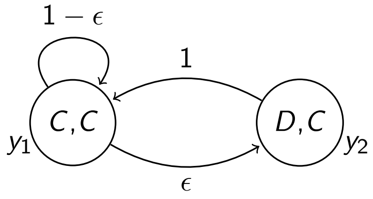

Figure S9: Product of the automata encoding Forgiver (S14) and ALLC (S26).

and $y_2 = \epsilon/(1+\epsilon)$. Hence, the average payoff per round for Forgiver playing against ALLD is given by:

$$R_{S_{14}\times S_{26}} = \frac{1}{1+\epsilon}\cdot R_{S_{26}\times S_{26}} + \frac{\epsilon}{1+\epsilon}\cdot R_{S_1\times S_{26}} = b-\epsilon\cdot\left(b+1-\frac{3}{1+\epsilon}\right)\ . \tag{S7}$$

Similarly, the average payoff per round for ALLC vs. Forgiver is:

$$R_{S_{26}\times S_{14}} = \frac{1}{1+\epsilon}\cdot R_{S_{26}\times S_{26}} + \frac{\epsilon}{1+\epsilon}\cdot R_{S_{26}\times S_1} = \frac{b\cdot(1-2\epsilon)}{1+\epsilon} + \epsilon\cdot(b+1)\ . \tag{S8}$$

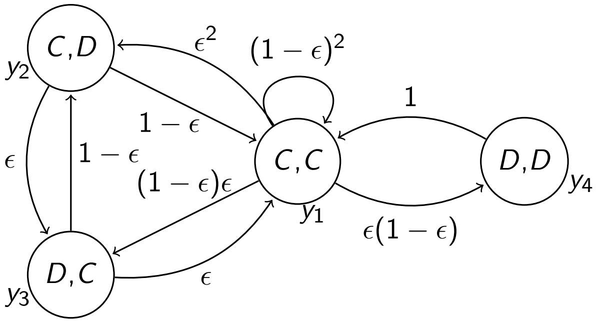

Figure S10: Product of the automaton encoding Forgiver (S14) multiplied with itself.

2.6 Forgiver vs. Forgiver

When Forgiver plays against itself we need to analyze an automaton with four states (Figure S10). We observe that in this obtained automaton the following equations hold:

$$\begin{aligned}
1 &= y_1 + y_2 + y_3 + y_4 \\
y_1 &= y_1 \cdot (1-\epsilon)^2 + y_2 \cdot (1-\epsilon) + y_3 \cdot \epsilon + y_4 \\
y_2 &= y_1 \cdot \epsilon^2 + y_3 \cdot (1-\epsilon) \\
y_3 &= y_1 \cdot \epsilon \cdot (1-\epsilon) + y_2 \cdot \epsilon \\
y_4 &= y_1 \cdot \epsilon \cdot (1-\epsilon)\ .
\end{aligned} \tag{S9}$$

After solving this system of equations (S9) we obtain for the frequencies of the states: $y_1 = 1/(1+3\epsilon-\epsilon^2)$, $y_2 = \epsilon/(1+3\epsilon-\epsilon^2)$, $y_3 = \epsilon/(1+3\epsilon-\epsilon^2)$, and $y_4 = (\epsilon-\epsilon^2)/(1+3\epsilon-\epsilon^2)$. Using the same approach as before, we calculate the average payoff per round for Forgiver vs. Forgiver as:

$$\begin{aligned}
R_{S_{14}\times S_{14}} &= \frac{1}{1+3\epsilon-\epsilon^2} \cdot R_{S_{26}\times S_{26}} + \frac{\epsilon}{1+3\epsilon-\epsilon^2} \cdot R_{S26\times S1} + \frac{\epsilon}{1+3\epsilon-\epsilon^2} \cdot R_{S1\times S26} \\
&\quad + \frac{\epsilon-\epsilon^2}{1+3\epsilon-\epsilon^2} \cdot R_{S1\times S1} = \\
&= 2b - 1 - \frac{(b-1)(1+7\epsilon)}{1+3\epsilon-\epsilon^2} + \epsilon \cdot (b-1)\ .
\end{aligned} \tag{S10}$$

3 Implementation of errors

In contrast to the works of Nowak and Sigmund (1994) and Frean (1994) where a strategy is defined by a quadruple (p_1, p_2, p_3, p_4), our strategies are defined by deterministic finite state

automata (Hopcroft et al., 2006) with one or two states. Their works incorporate errors directly in the four probabilities of a strategy. This type of errors is also known as errors in implementation (Sigmund, 2009). In our work we use imperceptive implementation errors. These are not included in the definition of a strategy and are generated externally. The difference between those two implementations being that in our work the player initiated the error does not observe the mistake (i.e., the player does not change his state according to the last erroneous move) whereas in the works of Nowak and Sigmund (1994) and Frean (1994) the erroneous player does observe the mistake (i.e., the player changes his state always according to the given probabilities). The imperceptibility of the implementation errors is related to perception errors described in Sigmund (2009).

References

M. R. Frean. The prisoner's dilemma without synchrony. *Proceedings of the Royal Society of London*, 257(1348):75–79, 1994.

J. E. Hopcroft, R. Motwani, and J. D. Ullman. *Introduction to Automata Theory, Languages, and Computation*. Prentice Hall, 3rd edition, 2006.

M. A. Nowak and K. Sigmund. The alternating prisoners dilemma. *Journal of Theoretical Biology*, 168:219–226, 1994.

K. Sigmund. *The calculus of selfishness*. Princeton University Press, 2009.
